# An Untethered Soft Robotic Dog Standing and Fast Trotting with Jointless and Resilient Soft Legs

**DOI:** 10.3390/biomimetics8080596

**Published:** 2023-12-08

**Authors:** Yunquan Li, Yujia Li, Tao Ren, Jiutian Xia, Hao Liu, Changchun Wu, Senyuan Lin, Yonghua Chen

**Affiliations:** 1 Shien-Ming Wu School of Intelligent Engineering, South China University of Technology, Guangzhou 510641, China; jiutian_xiajt@163.com; 2 School of Mechanical and Electrical Engineering, Chengdu University of Technology, Chengdu 610059, China; yujiali@swpu.edu.cn; 3 School of Mechatronic Engineering, Southwest Petroleum University, Chengdu 610500, China; 4 Department of Mechanical Engineering, The University of Hong Kong, Hong Kong SAR 999077, China; u3575051@connect.hku.hk (H.L.); u3575077@connect.hku.hk (C.W.); lsunkey@connect.hku.hk (S.L.); yhchen@hku.hk (Y.C.)

**Keywords:** untethered system, soft robot, quadruped robot, trotting gait, impact resistance

## Abstract

Soft robots are compliant, impact resistant, and relatively safe in comparison to hard robots. However, the development of untethered soft robots is still a major challenge because soft legs cannot effectively support the power and control systems. Most untethered soft robots apply a crawling or walking gait, which limits their locomotion speed and mobility. This paper presents an untethered soft robot that can move with a bioinspired dynamic trotting gait. The robot is driven by inflatable soft legs designed on the basis of the pre-charged pneumatic (PCP) actuation principle. Experimental results demonstrate that the developed robot can trot stably with the fastest speed of 23 cm/s (0.97 body length per second) and can trot over different terrains (slope, step, rough terrain, and natural terrains). The robotic dog can hold up to a 5.5 kg load in the static state and can carry up to 1.5 kg in the trotting state. Without any rigid components inside the legs, the developed robotic dog exhibits resistance to large impacts, i.e., after withstanding a 73 kg adult (46 times its body mass), the robotic dog can stand up and continue its trotting gait. This innovative robotic system has great potential in equipment inspection, field exploration, and disaster rescue.

## 1. Introduction

Soft robots are made of soft insulating materials and offer remarkable advantages in dealing with uncertain and complex environments compared with traditional hard robots [1,2]. Their soft body enables operation in confined spaces. Soft robots have been applied in different areas, such as industrial robots [3,4], wearable devices [5], and mobile robots [6,7]. Most soft mobile robots move on land and need to be connected to heavy pneumatic pumps or power supplies through tethers [8,9,10]. Developing untethered soft robots for terrestrial locomotion is still a major challenge because a great force or stiffness of the soft legs is required to transport mass [11].

Researchers have designed untethered soft mobile robots by utilizing different actuation methods. For pneumatically actuated soft robots, small pump systems are integrated into scaled-up robots with large actuation forces. Onal and Rus [12] designed a soft robot snake with a speed 19 mm/s, and Duggan et al. [13] developed an inchworm-inspired untethered soft robot with a crawling speed of about 0.28 mm/s.

Gas combustion can instantly release a large amount of energy. Tolley et al. [14] and Bartlett et al. [15] developed untethered jumping soft robots actuated by combustion that could jump off the ground for 0.6–0.76 m, nearly six times their body heights.

For other ways of actuation, researchers have developed untethered soft mobile robots by integrating compact batteries and microcontrollers in robots [16,17]. Huang et al. [18,19] designed shape memory alloy untethered soft robots, i.e., a legged robot that can walk at the speed of 3.2 cm/s and a caterpillar robot that crawls at the speed of 7.4 cm/s. Cao et al. [20] created an untethered soft robot using a dielectric elastomer actuator and simulated the locomotion of an inchworm; the robot can crawl at the speed of 4.16 mm/s. He et al. [21] proposed an untethered-legged soft robot actuated by liquid crystal elastomer (a thermoactive material) tubular actuators that can move at the speed of 0.04 mm/s.

Trotting gait is a moderate running dynamic gait for most quadruped mammals and is also the most efficient gait of all dog gaits [22,23]. During a trotting gait, the diagonal motion pattern provides mechanical stability for the quadruped body [24]. Researchers have developed a large number of advanced quadruped robots with hard mechanical linkages, and most of them used trotting gait for a stable and moderate speed dynamic locomotion, i.e., Bigdog [25], Spotmini [26], cheetah-cub [27], Hy-Q [28], and Tekken 2 [29]. Some researchers developed 3D-printed fully compliant/soft legs to achieve flexible locomotion of quadruped robots, such as [30,31], that demonstrated the feasibility of the compliant/soft legs in quadruped robots.

We aim to develop an untethered terrestrial mobile quadruped soft robot that can perform the trotting gait so it can improve its locomotion speed and mobility while maintaining its resilience and compliance advantages over hard robots. In our previous research, we proposed a new type of untethered soft actuator named the pre-charged pneumatic (PCP) soft actuator [32]. We developed soft robotic grippers [33,34], an amphibious robotic dog [35], and an untethered bioinspired quadrupedal robot that can walk [36]. The PCP soft actuator system is ideal for making untethered soft robots.

**Figure 1:**
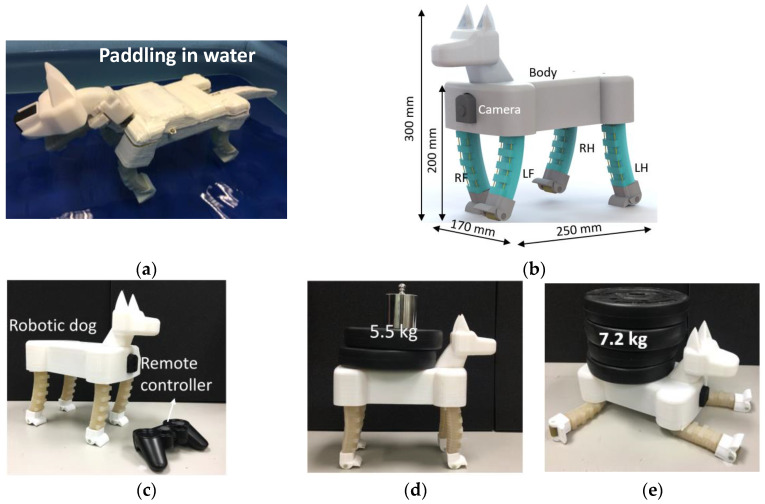
The proposed robotic dog. (**a**) Soft robotic dog paddling in water [33]. (**b**) 3D model of the new soft robotic dog design. (**c**) Deformation of legs when landing from a free fall of 20 cm height. (**d**) Carrying a static load of 5.5 kg. (**e**) Extreme deformation of legs under heavy weight.

In our previous research [35], we developed an amphibious soft robotic dog with four PCP soft actuators as legs (as shown in Figure 1a), and the paddling ability in an aquatic environment (swimming pool) was tested and proved feasible, with about 2 cm/s paddling speed. The on-land trotting gait was also designed and implemented on the amphibious soft robotic dog; however, in our previous research, only preliminary analysis and tests were conducted to demonstrate its on-land locomotion ability with an intuitively designed trotting gait, e.g., trotting on a flat terrain with fixed speed. In this research, we mainly focus on designing, modeling, implementation, and testing the trotting gait of the soft robotic dog in detail, such as how the trotting gait can be designed for a soft robotic dog with kinematic analysis, as well as comprehensively evaluating its on-land locomotion ability in different terrain conditions, including the load-carry ability, locomotion speed, and terrain adaptability. The overall dimensions (Figure 1b) of the robotic dog are 300 mm in height, 170 mm in width, and 250 mm in length. The legs are marked based on their positions: left front (LF), right front (RF), left hind (LH), and right hind (RH). The robot is integrated with power, a 720 p wireless camera and control system inside the body with a mass of 1.57 kg, and a gamepad that can be used for remote locomotion control (Figure 1c). An onboard pump or compressor of any kind is not applied. Our actuation method is essentially different from a motor tendon-driven soft robot; that is, the servomotor is not directly involved in the interaction with the environment, and the pre-charged pneumatic pressure is the one doing the work. Stiffness, force output, and impact resistance of the leg are mainly decided by the pre-charged pressure and elastomer material properties of the soft legs. The servomotors tension the tendon to help store elastic potential energy in the PCP soft legs, which are more elastic and resilient compared with the rigid robotic legs. The robotic dog with completely soft legs demonstrates good comprehensive performance in different tests summarized as follows:

1. The trotting speed can be controlled. The fastest trotting speed can reach 23 cm/s (0.9 body length/s), which is one of the fastest untethered terrestrial soft robots.
2. The robotic dog has a large load-to-weight ratio, i.e., the largest static load can be up to 5.5 kg (Figure 1d), and the largest load during locomotion can be 1.5 kg (nearly the same as the body mass).
3. The robotic dog’s trotting gait shows compliance and robustness. It can trot through different terrain conditions without feedback, and it can trot even when a 1.5 kg load is suddenly added to its back.
4. The robotic dog shows damage resistance to large impact during trotting, as shown in Figure 1e. In the follow-up experiment, even when a person (mass is nearly 50 times that of the robot) stands on the robotic dog, the soft legs experience extreme deformation, and the mobility of the robotic dog is not damaged.

## 2. Mechanical Design and Modeling of the Robot

### 2.1. Design of the Soft Leg

**Figure 2:**
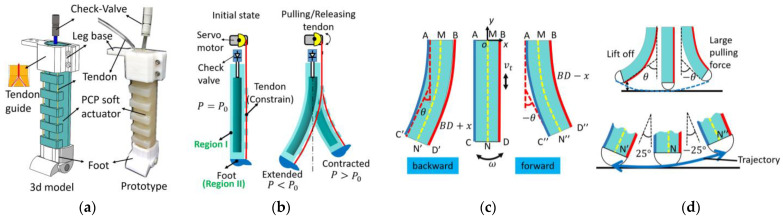
PCP soft leg. (**a**) PCP soft leg 3D model and prototype. (**b**) Motion diagram of the PCP soft leg. (**c**) Parameters during bending process of PCP soft actuator. (**d**) Bending range analysis and desired trajectory for robotic dog trotting gait.

The 3D model and prototype of the PCP soft leg are shown in Figure 2a. The main part of the PCP soft actuator is completely made of silicone rubber. The 3D-printed leg base and foot are installed on two ends of the PCP soft actuator for connection and support. The PCP soft leg has some key components such as a check valve and tendons, which distinguish it from other existing pneumatic or tendon-driven soft legs. With the check valve, the PCP soft actuator does not need to be connected to a pump; the pre-charged pressure can be changed accordingly to adjust the force output and leg stiffness. The tendon passes through the tendon guide and converges in the leg base through the tendon guide inside the base.

Figure 2b shows the schematic of the PCP soft leg’s movement. We charged an initial pressure $P_{0}$ (suggested value: $60\;kPa\leq P_{0}\leq 100\;kPa$) to the straight-state PCP soft leg. When the tendon is pulled from a straight state, the actuator contracts and bends to the right-hand side, and the pressure inside the chamber becomes larger than $P_{0}$. After the tendon is gradually released, the PCP soft actuator bends to the left-hand side, and the inner pressure becomes smaller than $P_{0}$. The largest right-hand side bending angle is decided by the servomotor rotation angle, and the largest left-hand side bending angle (when the tendon is completely released) is decided by the initial pressure $P_{0}$. When obstacles are present during the release of the tendon, the stored potential energy interacts with the obstacles, and the servomotor only acts as a linear constraint to control the pneumatic energy release rate.

Although geometrical modeling of the PCP soft actuator has been discussed in our previous research, those applications mainly analyzed the relationship between the bending angle and pulling-tendon distance, that is, the curve AC and BD of region I in Figure 2b,c, where AC represents the inextensible layer, and BD is the extensible deformation layer. For the application of the PCP soft actuator in the proposed robotic dog whose legs are installed vertically for trotting gait, we analyzed the movement of the actuator end CD of region II.

In Figure 2c, the change of the tendon length is defined as x, and the corresponding bending angle is defined as ±θ (θ is in the unit of radian). The length of the PCP soft leg A to B is defined as $l_{AB}$. Similarly, the length of A to C is defined as $l_{AC}$, and the length of A to M is defined as $l_{AM}$. Thus,

(1) $$x=\theta \cdot l_{AB}$$

The tendon-length change rate (tendon speed $v_{tendon}$) is linearly proportional to the bending angle θ change (bending speed).

(2) $$v_{tendon}=\frac{dx}{dt}=l_{AB}\cdot \frac{d\theta }{dt}=l_{AB}\cdot \omega$$

Given that the actuator end CD is not always parallel to the ground during bending, we need to design a foot by considering its contact with the ground. The trajectory equations N ($N_{x}$, $N_{y}$) (N is the middle point of CD, $N_{x}$, $N_{y}$ are the coordinate value of point N) in the bending process in coordinate xoy is as follows:

(3) $$N_{x}=\left\{\begin{array}{c} \begin{array}{c} -{sin}^{2}\left(\frac{\theta }{2}\right)\left(2\left(\frac{l_{AC}}{\theta }\right)+l_{AM}\right),\;\;\;\;\;\mathrm{bending}\;\mathrm{backward}\\ {sin}^{2}\left(\frac{\theta }{2}\right)\left(2\left(\frac{l_{AC}}{\theta }\right)-l_{AM}\right),\;\;\;\;\;\mathrm{bending}\;\mathrm{forward} \end{array}\\ 0,\;\;\mathrm{straight}\;\mathrm{state} \end{array}\right.$$

The original length of the PCP soft leg M to N is defined as $l_{MN}$.

(4) $$N_{y}=\left\{\begin{array}{c} -\sin\left(\frac{\theta }{2}\right)\cos\left(\frac{\theta }{2}\right)\left(2\left(\frac{l_{AC}}{\theta }\right)+l_{AM}\right),\;\;\;\mathrm{bending}\;\mathrm{backward}\\ -\sin\left(\frac{\theta }{2}\right)\cos\left(\frac{\theta }{2}\right)\left(2\left(\frac{l_{AC}}{\theta }\right)-l_{AM}\right),\;\;\;\mathrm{bending}\;\mathrm{forward}\\ -l_{MN},\;\;\mathrm{straight}\;\mathrm{state} \end{array}\right.$$

The trajectory of the foot is plotted in Figure 2d. When the backward bending angle ranges in $\theta \in \left[0{}^{\circ},25{}^{\circ}\right]$, the difference in *y*-axis values is approximately 1 mm. However, when the backward bending angle exceeds 25°, the difference increases, and the foot is lifted off the ground, causing the body to move downward. As a result, the shift between the gravity line and the support point becomes too large, and the soft leg can hardly support the body. For locomotion stability, the bending backward angle is limited to 25°. For bending forward, lift-off can provide compliance when trotting (i.e., climbing over obstacles). However, if the foot lifts off too much, the pulling force will be too large, and the leg may even buckle sometimes. In the gait design, the bending angle on each side is limited to the range of +25° to −25°.

### 2.2. Design of the Robotic Dog

**Figure 3:**
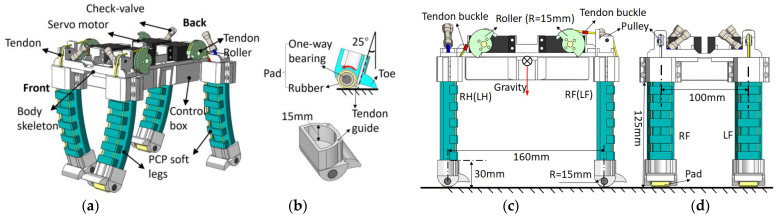
Detailed design parameters of the robotic dog. (**a**) Mechanical components inside the body shell. (**b**) Design of the foot. (**c**) Side view of the robotic dog. (**d**) Front view of the robotic dog.

Figure 3a,b show details of the robotic dog design. The tendon roller is in the shape of a half circle (radius of 15 mm). Two pulleys are designed above the RF and LF two front legs’ bases, and the tendon passes through the pulley and connects with the tendon roller (Figure 3a). For the two hind legs (LH and RH), the tendon is directly connected to the roller. The gravity center is located in the middle of the torso. Figure 3b shows the detail of the foot design based on the geometrical analysis of the PCP soft actuator. The foot is attached to the end of the PCP soft actuator, and the tendon passes through the foot’s tendon guide. The pad of the foot is made of a one-way bearing with a rubber outer layer to increase the friction force. When the leg bends backward, the one-way bearing locks up and provides static friction force, so the entire leg rotates along the contact point and brings the body forward. When the leg is pulled to bend forward and lift off the ground, the bearing can roll freely in this direction to reduce the friction force. A toe is designed as a support to prevent further leg rotation along the contact point. A tendon buckle is fixed on the tendon section (Figure 3c) to function as a mechanical lock when the leg backward bending angle reaches 25° to reduce the effect on the servomotor at this extreme position as the servomotor changes its rotation direction at this point. The front view of the robotic dog is shown in Figure 3d.

## 3. Trotting Gait Design and Analysis

Trotting gait is often seen in nature, has good energy efficiency with different speeds, and features no significant pitch or roll motion during each stride [28]. In this section, we present the design of the trotting gait and analyze the gait parameters.

### 3.1. Kinematic Analysis of a Soft Leg in One Motion Cycle

**Figure 4:**
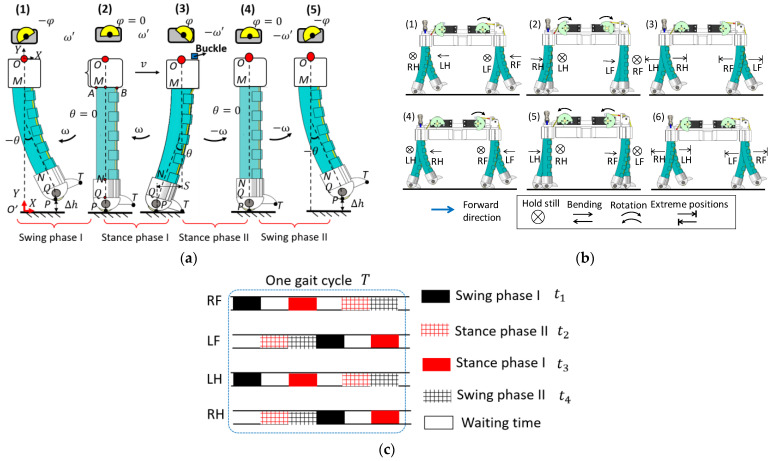
Trotting gait design of the robotic dog. (**a**) Kinematic analysis of the robotic dog’s leg during one cycle of motion. (**b**) Trotting gait decomposition diagram. (**c**) Trotting gait control pattern.

To design the trotting gait of the robotic dog, we first analyzed the kinematic model of a soft leg during one motion cycle. For one leg during the trotting gait, two phases must always be present [27]. The stance phase usually means the leg is supporting the body and pushing the body forward, and the swing phase means the leg is in the air and swinging forward for the next stance. For the proposed soft leg, we have slightly modified the gait-phase division: stance phase I and stance phase II, swing phase I, and swing phase II. The leg is segmented into three parts (OM, MN and NP) along its vertical centerline for analysis (Figure 4a(2)). The length of the leg base (*l*_OM_) and foot (*l*_NP_) are fixed, and the length of the PCP soft actuator (*l*_MN_) changes during a gait stride. 

First, the foot is lifted away from the ground for height ∆h. The swing phase I starts from Figure 4a(1),(2), during which the servomotor starts to release the tendon and the leg bends from −25° to 0°, and the foot pad touches the ground at point P, as shown in Figure 4a(2). The tendon is further released, and then the stance phase I occurs as defined in Figure 4a(2),(3). The leg continues to bend from 0° to +25°. The entire leg rotates along contact point P until the foot toe touches the ground at point T (Figure 4a(3)). At the same time, a metal buckle on the tendon blocks the base and prevents the leg from further bending. During the stance phase I, the leg base moves forward for distance S, which is defined as a stride length. The stance phase II is shown in Figure 4a(3),(4). The servomotor starts to pull the tendon to contract the bent soft leg, the foot toe T leaves the ground, and the leg bends forward from 25° to 0°. During this phase, the friction force between the pad and the ground can be ignored because the pad is a one-way rotation mechanism. As a result, the base OM does not move in the stance phase II. Figure 4a(4),(5) is the swing phase II. The tendon is further pulled, the leg bends forward from 0° to −25°, and the foot is lifted away from the ground for ∆h.

The bending angle is related to the servomotor rotation angle, given as

(5) $$\theta =\frac{\varphi R_{roller}}{l_{AB}},$$

where *R*_roller_ is the radius of the tendon roller that installed on the servo motor, *φ* is the rotation angle of the servo motor.

The angular speed is related to servomotor rotation speed $\omega^{\prime }$,

(6) $$\omega =\frac{\omega^{\prime R_{roller}}}{l_{AB}}.$$

Substituting R_roller = 15 mm and l_AB = 20 mm into (5) can obtain the range of servomotor rotation angle.

We have added the base OM and the foot NP to the Equations (3) and (4); the trajectory of the foot pad’s lowest point P in leg base coordinate *XOY* can be represented by $\vec{OP}=\left[P_{X},P_{Y}\right]$, with

(7) $$P_{X}=\left\{\begin{array}{c} \begin{array}{c} -{sin}^{2}\left(\frac{\theta }{2}\right)\left(2\left(\frac{l_{AC}}{\theta }\right)+l_{AM}\right)-l_{NQ}\sin\left(\theta \right),\;\;\mathrm{bend}\;\mathrm{backward}\\ {sin}^{2}\left(\frac{\theta }{2}\right)\left(2\left(\frac{l_{AC}}{\theta }\right)-l_{AM}\right)+l_{NQ}\sin\left(\theta \right),\;\;\mathrm{bend}\;\mathrm{forward} \end{array}\\ 0,\;\;\mathrm{straight}\;\mathrm{state} \end{array}\right.$$

(8) $$P_{Y}=\left\{\begin{array}{c} -\left[l_{OM}+sin\left(\frac{\theta }{2}\right)\cos\left(\frac{\theta }{2}\right)\left(2\left(\frac{l_{AC}}{\theta }\right)+l_{AM}\right)+l_{NQ}cos\left(\theta \right)+l_{QP}\right],\\ \mathrm{bend}\;\mathrm{backward}\\ -\left[l_{OM}+\sin\left(\frac{\theta }{2}\right)\cos\left(\frac{\theta }{2}\right)\left(2\left(\frac{l_{AC}}{\theta }\right)-l_{AM}\right)+l_{NQ}cos\left(\theta \right)+l_{QP}\right],\\ \mathrm{bend}\;\mathrm{forward}\\ -l_{OP},\;\;\;\mathrm{straight}\;\mathrm{state} \end{array}\right.$$

where θ=ωt, ω is the bending speed of the soft actuator, t is the time interval of each phase, $l_{NQ}$ is the length of N to Q, and $l_{OP}$, $l_{OM}$ and $l_{QP}$ are defined in the same way.

Basing on the trajectory of the foot pad P in coordinate *XOY*, we can obtain the base trajectory *O* in coordinate *XO*′*Y* by coordinate transformation. The foot does not come into contact with the ground in the swing phases I and II or generate forward force in the stance phase II; therefore, the base *O* trajectory $\vec{O^{\prime }O}=\left[O_{x},O_{y}\right]$ should be represented based on different gait phases:

(9) $$O_{X}=\left\{\begin{array}{c} 0\;\;\;\mathrm{swing}\;\mathrm{phase}\;I\\ -P_{X}\left(bend\;backward\right)\;\mathrm{stance}\;\mathrm{phase}\;I\\ 0\;\;\;\mathrm{stance}\;\mathrm{phase}\;\mathrm{II}\\ 0\;\;\;\mathrm{swing}\;\mathrm{phase}\;\mathrm{II} \end{array}\right..$$

(10) $$O_{Y}=\left\{\begin{array}{c} OO^{\prime }+P_{Y}\left(bend\;backward\right)\;\;\mathrm{swing}\;\mathrm{phase}\;I\\ OO^{\prime }\;\;\;\;\mathrm{stance}\;\mathrm{phase}\;I\\ OO^{\prime }\;\;\;\mathrm{stance}\;\mathrm{phase}\;\mathrm{II}\\ OO^{\prime }+P_{Y}\left(bend\;forward\right)\;\;\;\mathrm{swing}\;\mathrm{phase}\;\;\mathrm{II} \end{array}\right.$$

The stride length S and foot lift-off height ∆h can be calculated from (9) and (10):

$$\begin{array}{c} S=O_{X}\left(stance\;phase\;I\right)\approx 32\;mm\\ ∆h=O_{Y}\left(swing\;phase\;II\right)\approx 8\;mm \end{array}$$

In stance phase I, the bending angle is about 25; in swing phase II, the bending angle is about −25°. In the prototype test, the stride length *S* and lift-off height ∆h are larger than the calculated result, S≈30 mm and ∆h≈12 mm, due to manufacturing errors and uneven deformation of the PCP soft legs.

### 3.2. Trotting Gait Design

A complete gait-cycle diagram is demonstrated in Figure 4b, and the gait control pattern is demonstrated in Figure 4c. The control signals are directly given to the servomotors through a commercially available servomotor controller, which can control the rotation angle and time interval of four servomotors.

Figure 4b shows that from (1) to (2), the RH and LF legs hold still, and the LH and RF legs are in the swing phase I. After the LH and RF legs touch the ground, they hold still and provide static friction force, waiting for the RH and LF legs to bend forward (stance phase II). From (2) to (3), all four legs are in the straight state. The LF and RH legs bend forward (swing phase II) and lift off the ground. At the same time, the RF and RH legs bend backward (stance phase I) and push the robotic dog forward. The four legs reach extreme positions at (3), where the LH and RF legs’ bending angles are 25°, and those of the RH and LF legs are −25°. Step (3) is the end of half a trotting gait cycle; from (1) to (3), the LH and RF legs push the body forward. Steps (4) to (6) have the opposite gait pattern compared with steps (1) to (3).

The time of a gait cycle is defined as T (Figure 4b). The extreme positions of the legs are fixed (±25°), indicating that the maximum gait stride length during one gait cycle is 2S. We define $t_{1},t_{2},t_{3},\;\mathrm{and}\;t_{4}$ as the time intervals of swing phase I, stance phase II, stance phase I, and swing phase II ($t_{3}=t_{4}$), respectively. According to Figure 4c, $T=2t_{1}+2t_{2}+2t_{3}$. The average trotting speed $\bar{v}$ can be calculated as

(11) $$\bar{v}=\frac{2S}{T}=\frac{S}{t_{1}+t_{2}+t_{3}}.$$

The servomotor extreme rotation angle of these four phases is the same in the planned forward trotting gait. Therefore, the trotting speed can be controlled directly only by adjusting the time interval of different phases. The turning locomotion can be realized by adjusting the stride length of the related legs on the side where the dog plans to turn.

## 4. Experimental Test and Discussion of PCP Soft Legs and the Robotic Dog

### 4.1. Experimental Test of the Pulling Force and Reaction Time of the PCP Soft Legs

A PCP soft-leg prototype was fabricated for experimental tests. The fabrication process of the PCP soft leg can be found in a previous work [32]. The materials used for the PCP soft leg are listed below for reproduction: silicone rubber SHORE-A 20D (supplier: yuanmuzigongyi, Shenzhen, Guangdong, China), check-valve AKH04-00 (back pressure 1.5 MPa, cracking pressure 0.005 MPa, supplier: shuanglin, Wenzhou, Zhejiang, China), and tendon (nylon, supplier: kastking, Dongguan, Guangdong, China).

The PCP actuator’s largest bending angle is determined by its pre-charged air pressure obtained through the experimental test. When the pre-charged air pressure is 40 kPa, the largest bending angle is smaller than 25°; therefore, the pre-charged air pressure must be at least 40 kPa. In this research, the pressure for our analysis and tests was set at 60, 80, and 100 kPa. To select the servomotor we required, we needed to measure the tendon force during the PCP soft-actuator bending. We measured the tendon force F_T_ versus the pulling distance using different values of pre-charged air pressure P0, and the modeling curve can be plotted directly based on the analysis in [30].

**Figure 5:**
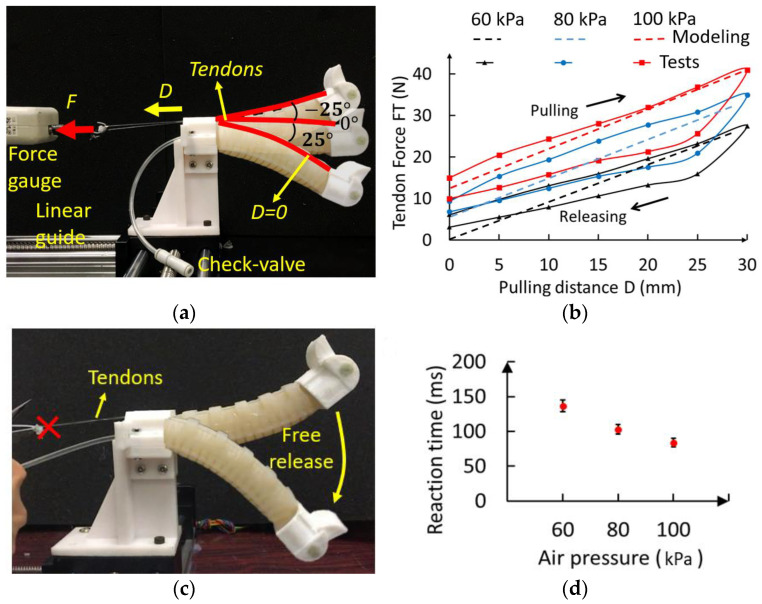
Measurement of the PCP soft-leg prototype. (**a**) Experimental setup of the tendon force vs pulling distance. (**b**) Tendon force under different pre-charged pressure. (**c**) Experimental set up of releasing time measurement. (**d**) Reaction time of PCP soft actuator with different pre-charged pressure.

The experiment setup is shown in Figure 5a. A force gauge was installed on a linear guide to pull the tendon of the PCP soft actuator. The pulling distance is defined as 0 when the bending angle is 25° and reaches a maximum value when bending to −25°. The relationship between the tendon force (F_T_) and pulling distance (D) and the modeling results are plotted in Figure 5b. According to the experiment results, the largest forces required to pull the actuator to −25° are about 40 N for 100 kPa, 30 N for 80 kPa, and 20 N for 60 kPa (the servomotor we used has a stall torque of 250 Ncm). As shown in Figure 5c, we also measured the reaction time of the soft actuator when the tendon is freely released. The actuator is pulled to −25°, and the tendon is cut. The fast bending of the PCP soft actuator was recorded with a high-speed camera, and the video was then analyzed with the open-source motion analysis software Kinovea-0.9.4 version. We measured the first time that it takes the actuator bend to another extreme position. The experiment results are shown in Figure 5d. The reaction time of the PCP soft actuator under different values of pre-charged pressure are: 140 ms in 60 kPa, 100 ms in 80 kPa, and 80 ms in 100 kPa. Therefore, the fastest servomotor rotation speed ($\frac{0.12s}{60}{}^{\circ}$) is smaller than the free-releasing bending speed. This finding indicates that the tendon always constrains the bending motion during the release. As a result, the bending speed of the PCP soft leg is determined by the servomotor rotation speed.

### 4.2. Experimental Test of the Load-Carrying Abilities of the PCP Soft Legs

**Figure 6:**
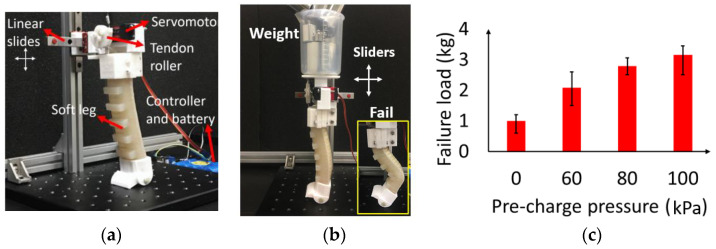

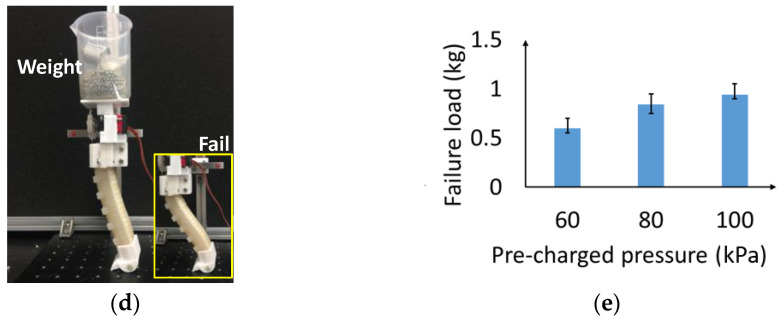
Experimental test. (**a**) Test platform of the PCP soft leg. (**b**) Buckling failure load test at straight state. (**c**) Results of the failure load test at straight state. (**d**) Buckling failure load test at bending 20° state. (**e**) Results of the failure load test at bending 20° state.

We built a test platform for measuring the load-carrying ability of the PCP soft leg (Figure 6a). A PCP soft-leg module with a servomotor fixed on the leg base was designed for this test, and the PCP soft-leg module was installed on a pair of linear guides. During trotting gait, the robotic dog body seldomly rotates along the base, and the linear guides can only move freely in vertical and horizontal directions to simulate the movement of one PCP soft leg. The mass of the test platform (includes the servomotor, leg base, and two linear guides) is already around 400 g, and this fact should be taken into consideration after the tests.

To measure the largest load that the robotic dog can carry during trotting gait without falling, we conducted experimental tests to measure the vertical load that causes the buckling failure of the soft leg at a standing straight state (bending angle 0°) and bending state (bending angle 25°), as shown in Figure 6a,b. The PCP soft-leg module is fixed on the linear guides, and a beaker is mounted on the top of the leg module for holding different weights. The mass of the robotic dog is around 1.5 kg; therefore, in trotting gait, the body weight divided by each leg is 0.75 kg. To test the net weight that a leg can carry, we have added 350 g weight to the beaker as a base weight. Therefore, the total weight of the test module becomes 0.75 kg.

We tested the soft leg at its standing straight state with different values of pressure $P_{0}$. The weights were then added gradually to the leg module in units of 100 g. We measured the largest weight a leg can hold before buckling failure (the leg loses balance). This experiment reveals the largest load that the robotic dog can carry at the standing straight state. For each pressure, we conducted the failure test 10 times, and the results are plotted in Figure 6c. The soft leg with pre-charged pressure has a larger failure load (100 kPa, 2.5–3.5 kg) than the non-pre-charged soft leg (0 kPa, 0.5–1.2 kg). The failure load value has such a large range because of the three modes of buckling failure: the first is when the foot goes in front of the weight (smallest failure load), the second is when the foot remains at the initial position (largest load), and the third is when the weight goes in front of the foot. The failure load is also significantly positively related to the pre-charged pressure. At the bending state (Figure 6d), the failure load experiment reveals the minimum weight that a leg can carry during a trotting gait. The failure load that can be held by one leg is 0.6, 0.7, and 0.9 kg at 60, 80, and 100 kPa, respectively, as shown in Figure 6e. Therefore, the largest load at the trotting gait that can be carried by the robotic dog can be estimated as 1.2 (60 kPa), 1.4 (80 kPa), and 1.8 kg (100 kPa).

### 4.3. Experimental Test of the Trotting Gait Controllability and Robustness of the Robotic Dog

**Figure 7:**
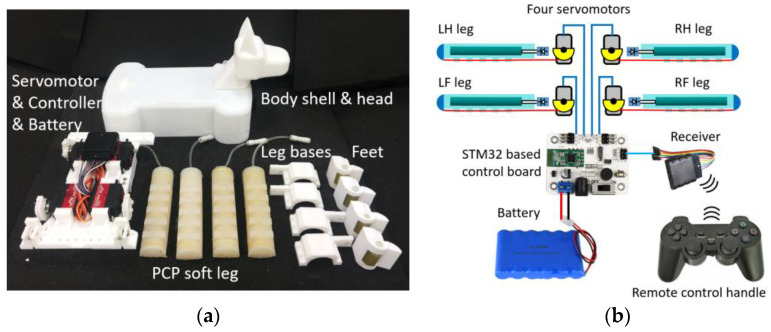
Robotic dog prototype. (**a**) Components of the robotic dog. (**b**) Control circuit diagram.

A robotic dog prototype was manufactured for experimental tests. Its components are shown in Figure 7a, including body shell and head; servomotor, controller, and battery; PCP soft legs; leg bases; and feet. The body shell and head, leg bases and feet, and the base installed by the servomotors are all 3D printed with Polylactic Acid (PLA) (3d printing filament, supplier: eSUN, Xiaogan, Hubei, China) materials. The four PCP soft legs are the same as the ones used in previous tests. The control circuit diagram is shown in Figure 7b. The control board is connected to a 7.4 V 6000 mAh Li-ion battery (supplier: shengyuan, Guangzhou, Guangdong, China), a wireless receiver, and four servomotors that drive four PCP soft legs. The controller is a Hiwonder 6-way Bluetooth controller(supplier: Hiwonder, Shenzhen, Guangdong, China), a commercially available controller (supplier: Hiwonder, Shenzhen, Guangdong, China) based on STM32 CPU integrated on board. The servomotor is Hiwonder LD-25MG (supplier: Hiwonder, Shenzhen, Guangdong, China) with 2.5 Nm stall torque connected with a 15 mm diameter tendon roller to ensure that it meets the requirement of driving the PCP soft legs. Through the receiver, the servomotors can be controlled with a remote-control handle.

Based on the modeling of the gait pattern and the trajectory of the foot shown in Figure 4a, the change in the rotation angle of the steering gear for one leg can be interpolated, as shown in Table 1. The robotic dog is open loop controlled based on a pre-programmed controlling sequence (Table 2), a programming software that ensures that the servomotor angle sequence control is provided with the control board. The program is implemented on the robotic dog prototype.

**Figure 8:**
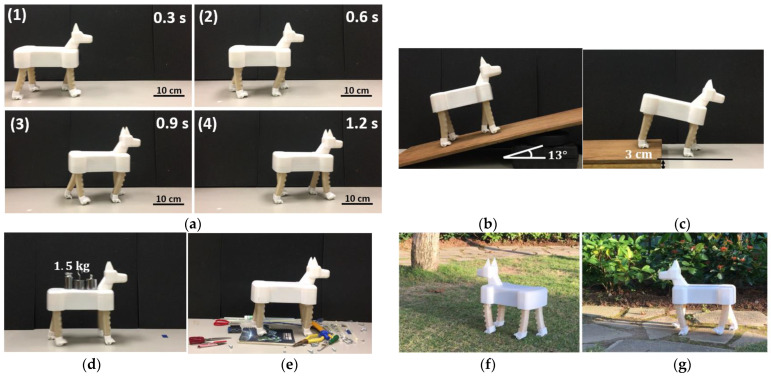
Trotting gait test in different conditions with open loop control. (**a**) Trotting gait demonstration. (**b**) Climbing a slope. (**c**) Trotting down a step. (**d**) Weight-carrying trotting. (**e**) Trotting through rough terrain. (**f**) Trotting on lawn. (**g**) Trotting on uneven stone road.

We tested the trotting speed at different gait cycle times to check the controllability of the robotic dog. The prototype trots along a straight line for a distance of 75 cm with two different gait cycle times T_1_ = 500 ms and T_2_ = 1000 ms, and the time intervals of each stance phase are 1/6 of the gait cycle, that is, 100 and 200 ms, respectively. During the tests, the ground was marked at a 75 cm distance with adhesive tape, and the trotting of the robotic dog was recorded by a camera. We analyzed the video through the software Kinovea, and the test results are summarized in Table 3. The calculated speed of *T*_1_ is *v*_1_ = 2*S*/*T*_1_ = 6 cm/0.5 s = 12 cm/s, and the measured speed at 60, 80, and 100 kPa is relatively 13.3, 14.2, and 15 cm/s, respectively. The calculated speed of *T*_2_ is *v*_2_ = 2*S*/*T*_1_ = 6 cm/1 s = 6 cm/s, and the speed at 60, 80, and 100 kPa is relatively 6.9, 7.3, and 7.7 cm/s, respectively. The reason why the actual speed is larger than the calculated speed is that in the stance phase I, the tendon cannot immediately completely stop the soft leg because of velocity inertia. As a result, the dog moves forward for a while after the servomotor stops rotating. The larger the pressure, the more evident the difference will be. When we set the trotting gait cycle to T ≈ 360 ms (shortest cycle for the servomotor) with 100 kPa pre-charged pressure (Figure 8a), the robotic dog can reach the fastest trotting speed of 23 cm/s or 0.8 km/h, about 0.98 body length per second. Whether in absolute speed or relative speed to body length, the robotic dog can move on flat terrain much faster than most existing untethered soft robots.

The trotting speed limitation is mainly decided by two factors: the servomotor rotation speed and the intrinsic bending speed of the PCP soft legs. The intrinsic bending speed of the PCP soft legs is decided by the pre-charged pressure: the larger the pre-charged pressure is, the faster the bending and trotting speeds will be. However, the bending of the PCP soft legs is controlled by the servomotors. If the servomotors’ rotation is slower than the bending of the PCP soft legs, then the speed is limited by the servomotor’s rotation speed. If the servomotor’s rotation is faster than the intrinsic bending of the PCP soft legs, then the speed is limited by the intrinsic bending speed of the PCP soft legs (i.e., pre-charged pressure). 

We tested the robotic dog’s trotting compliance and adaptability under different terrain conditions without feedback control. We set a moderate trotting speed (≈12 cm/s) in these tests; trotting too fast may cause instability. Tests in Figure 8b-d were conducted under different values of pre-charged pressure (60, 80, and 100 kPa). Under the same open loop control algorithms, the robotic dog can climb up a slope (Figure 8b, maximum slope angle 13°, 100 kPa), trot down a step (Figure 8c, maximum height of the step 3 cm, 23% of the leg length, 100 kPa), trot with 1.5 kg weight that is about 96% of its body weight (Figure 8d, body weight 1.57 kg (100 kPa)), and even carry 1.8 kg with an unstable trotting gait. It should be noted that the step height of the soft leg primarily depends on the extent of the tendon contraction. Climbing up higher steps can be achieved through the implementation of a larger steering wheel or by increasing the rotations of the servomotor to increase each step’s height. The robotic dog performs better with 100 kPa pre-charged pressure than with 60 kPa pressure when dealing with different situations. The reason for this could be that the robotic dog requires enough stiffness rather than compliance to overcome different terrain conditions. The robotic dog can trot on a flat laboratory floor and adapt to different terrain conditions, as shown in Figure 8e–g. It can also trot over the obstacles (height 1 cm, range 70 cm) with a success rate of 80% under laboratory conditions; the height and range dimensions describe the average dimensions of the obstacles on the ground. We define a successful pass as the robotic dog passing through obstacles without falling over or getting caught by the obstacles. For every trail, we placed the obstacle back in place. The robotic dog can also trot at a moderate speed (8–10 cm/s) on lawns and rough stone roads, proving the high terrain adaptability of the proposed design. A Supplementary Video S1 is provided for the tests in Figure 8.

### 4.4. Experimental Test of Robotic Dog Impact Resistance

Experiments were conducted to test the impact resistance of the robotic dog. We simulated some of the possible impacts in real application scenes such as being hit/pressed by heavy objects. The impacts were all vertical.

**Figure 9:**
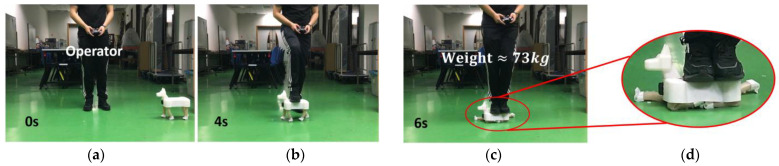

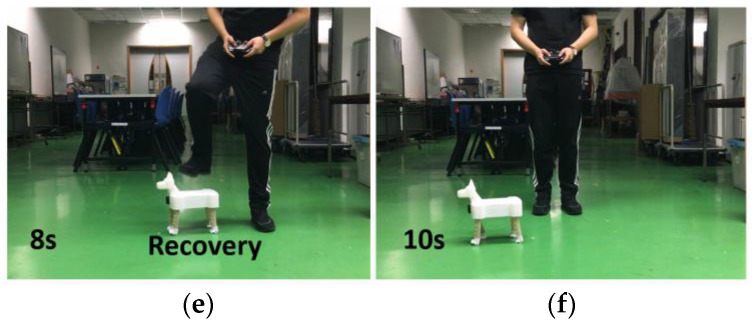
Damage resistance test. (**a**–**f**) Stepped on by a standing adult male (73 kg).

An impact test is shown in Figure 9. When the robotic dog trotting on flat terrain passes by the operator with a weight of 73 kg (Figure 9a,b), the operator steps on the robotic dog’s body until two feet are completely off the ground (Figure 9c,d). Although lasting for only some microseconds (≈400 ms, counting the time that both feet of the operator are lifting off the ground, analyzed by the software Kinovea-0.9.4 version), these soft legs withstand nearly 73 kg weight stepping over it without breaking or failing. As shown in the magnified view, the legs are extremely deformed. When the operator steps off the robotic dog and lifts the foot up as shown in Figure 9e, these legs rebound to the normal state in an extremely short time (~0.3 s) and without any extra adjustment, and then continue the trotting gait, as shown in Figure 9f. In the impact test, the PCP soft legs are working perfectly after these impacts and show no damage. As long as the body shell is strong enough, the robotic dog can resist extreme impact without losing its mobility. A Supplementary Video S2 is provided for the tests in Figure 9. A Supplementary Video S3 about the robotic dog navigating through the lab is provided as well. 

### 4.5. Comparison of the Robotic Dog with Related Work

The gait pattern and locomotion speed of the robotic dog is compared with other typical untethered soft mobile robots, as shown in Table 4. Most soft mobile robots adopt the crawling gait; therefore, their locomotion speed is limited, e.g., 8 out of 12 robots are slower than 0.1 BL/s (body length/s). Our robotic dog’s locomotion speed (0.98 BL/s) and gait pattern (trotting) are in a leading position compared with other untethered soft robots, as shown in Table 4.

**Figure 10:**
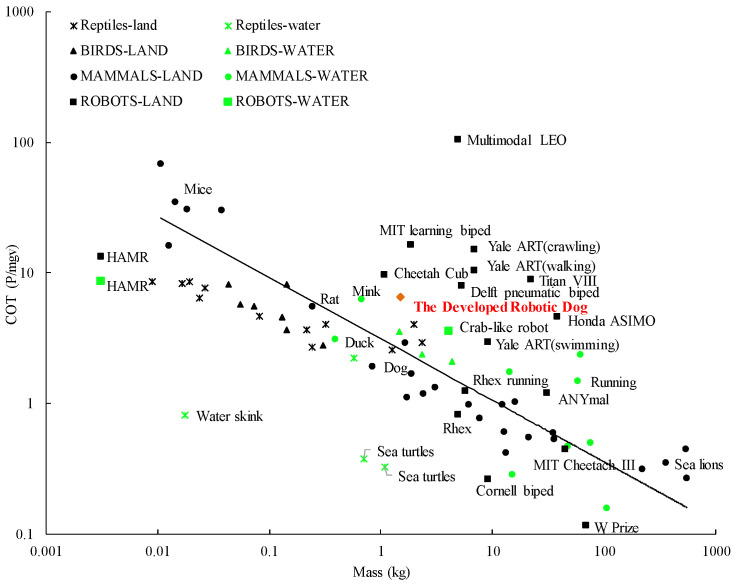
Proposed robotic dog situates itself well among other animals and robots, outperforming some state-of-the-art legged robots.

We also calculated the minimum cost of transport (COT) performance of the robotic dog based on [42] and compared it with the minimum COT of many reptiles, mammals, and legged robots (Figure 10). When moving on the ground, the minimum COT of the robotic dog without a load is 6.5625. The position of our robotic dog in this figure is quite close to the regression line, indicating that the COT/mass ratio is close to the average values of animals and other excellent mobile robots (including the hard robots). With a small body weight and low COT, the proposed robotic dog is a soft mobile robot with high mobility and low carbon cost.

## 5. Conclusions and Future Work

In this research, we present the design and trotting gait implementation of an untethered robotic dog with completely soft legs. This work is the first to report a design of an untethered quadruped robot that can trot dynamically with soft actuators, a feature rarely seen in untethered soft mobile robots. In the experimental test, the robotic dog demonstrates the trotting gait with the fastest trotting speed of up to 23 cm/s (0.98 body length per second) and the largest trotting payload of up to 1.5 kg (nearly 96% of body mass). In the standing state, the robotic dog can hold a weight of 5.5 kg (367% of body mass). With open loop control, the robotic dog can trot over different terrains (13° slope, 3 cm step, rough terrain, and natural terrains).

The robot prototype demonstrates the potential application of the PCP soft actuation system in untethered soft robots. With a clever combination of hard and soft robotics, the robotic dog exhibits greatly improved mobility and controllability while maintaining its compliance and damage resistance. The design concept might be able to promote the development of soft robotics in practical applications.

This robotic dog is still an initial prototype demonstrating a planar trotting gait with only four motors. Therefore, its mobility and locomotion speed still can hardly match today’s highly flexible quadruped hard robot. In the future, other motors can be used to develop a legged robot with a high degree of freedom, good controllability, and great compliance and damage resistance. Sensors such as gyroscopes or force sensors can be integrated into the legged robot for advanced and reliable control.

## Figures and Tables

**Table 1:** Servomotor angle of a leg’s one cycle of motion.

States in Figure 4a	A	B	C	D	E
Bending angle	−25°	0°	25°	0°	−25°
Servomotor angle	−30°	0°	30°	0°	−30°

**Table 2:** Trotting gait control signal.

Sequence	RF	LF	RH	LH	Time Interval
1	−30°	30°	30°	−30°	1 to 2: *t*_1_
2	0°	30°	30°	0°	2 to 3: *t*_2_
3	0°	0°	0°	0°	3 to 4: *t*_3_ (*t*_4_)
4	30°	−30°	−30°	30°	4 to 5: *t*_1_
5	30°	0°	0°	30°	5 to 6: *t*_2_
6	0°	0°	0°	0°	6 to 1: *t*_3_ (*t*_4_)

**Table 3:** Trotting speed with different gait cycle T.

Trotting Gait Cycle			Trotting Speed		
T = 2(*t*_1_ +*t*_2_ +*t*_3_)			60 kPa	80 kPa	100 kPa
T_1_ ≈ 500 ms			13.3 cm/s	14.2 cm/s	15 cm/s
*t*_1_ = 83 ms	*t*_2_ = 83 ms	*t*_3_ = *t*_4_ = 83 ms			
T_2_ ≈ 1000 ms			6.9 cm/s	7.3 cm/s	7.7 cm/s
*t*_1_ = 166 ms	*t*_2_ = 166 ms	*t*_3_ = *t*_4_ = 166 ms			

**Table 4:** Comparison of our robot with other untethered soft mobile robots.

Research	Actuation	Gait Type	Max Speed
[12]	Pneumatic	Crawling	1.9 cm/s (0.07 BL/s)
[14]	Pneumatic	Crawling	0.5 cm/s (0.007 BL/s)
[18]	SMA	Trotting	3.2 cm/s (0.56 BL/s)
[20]	DEA	Crawling	0.2 cm/s (0.02 BL/s)
[21]	LCE	Walking	0.004 cm/s (0.0005 BL/s)
[36]	DCPCP	Walking	10 cm/s (0.33BL/s)
[37]	Tendon	Trotting	4.3 cm/s (~0.22BL/s)
[38]	Pneumatic	Rolling	6 cm/s (~0.036 BL/s)
[39]	Pneumatic	Walking	(0.09 BL/s)
[40]	Pneumatic	Crawling	1.412 cm/s (0.082BL/s).
[41]	Pneumatic	Crawling	0.516 cm/s (0.0225BL/s)
Our robot	PCP	Trotting	23 cm/s (~0.98 BL/s)

BL/s: Body Length/s; SMA: Shape Memory Alloy; DEA: Dielectric Actuator; LCE: Liquid Crystal Elastomer; DCPCP: Double Chamber PCP.

## Data Availability

Data are contained within the article and Supplementary Materials.

## Supplementary Materials

The following Supporting Information can be downloaded at: https://www.mdpi.com/article/10.3390/biomimetics8080596/s1, Video S1: Robotic dog trotting on different terrains; Video S2: Robotic dog bearing heavy load and impact; Video S3: Robotic dog navigating through the lab.
